# Carriage Error Identification Based on Cross-Correlation Analysis and Wavelet Transformation

**DOI:** 10.3390/s120709551

**Published:** 2012-07-12

**Authors:** Donghui Mu, Dongju Chen, Jinwei Fan, Xiaofeng Wang, Feihu Zhang

**Affiliations:** 1 School of Mechanical & Electrical Engineering, Beijing University of Chemical Technology, Chao Yang District, Beijing 100029, China; E-Mails: badyun@163.com (D.M.); jwfan@bjut.edu.cn (J.F.); wxf19982000@163.com (X.W.); 2 Department of Mechanical Engineering, Harbin Institute of Technology, Harbin 15001, China; E-Mail: zhangfhu@hit.edu.cn

**Keywords:** carriage error, multi-body system, error identification, cross-correlation analysis, wavelet transform

## Abstract

This paper proposes a novel method for identifying carriage errors. A general mathematical model of a guideway system is developed, based on the multi-body system method. Based on the proposed model, most error sources in the guideway system can be measured. The flatness of a workpiece measured by the PGI1240 profilometer is represented by a wavelet. Cross-correlation analysis performed to identify the error source of the carriage. The error model is developed based on experimental results on the low frequency components of the signals. With the use of wavelets, the identification precision of test signals is very high.

## 1. Introduction

With the rapid advances in the development of electronic and optical devices, machines need to meet high precision requirements. Component errors of machine tools are the main factor which affects the machinery accuracy. Due to some limitation reasons, some errors cannot be tracked in real-time, hence identifying the main errors which affect machinery accuracy is important.

A carriage is an oriented-device that can travel in a given trajectory. It is one of the important moving parts which can determine the surface roughness, the surface shape and the relative position. It has a direct effect on the processing results. In order to improve the accuracy of a machinery tool, analyzing and identifing the carriage error(s) is essential.

The measurement of the carriage straightness error plays an important role in metrology. Various methods are adopted in the industrial measurement field [1–5]. Wei Gao [6] used the capacity probe and reversal method to measure and compensate the straightness of guideways. Other attempts were made to measure motion error sources of NC machines from circular tests. However, in the case of long-range measurement, the accuracy is affected by environmental conditions.

Some researchers have analyzed and identified the motion errors of the machine tool including the guideway errors using some direct ways. For example, Kakino *et al.* [7–9] proposed a step-by-step identification method of dominant motion error sources with the aim of extracting major motion error patterns from several circular test results. However, since the method requires an insight into motion error patterns step by step, it may be of limited use if error sources simultaneously influence the motion errors. Recently, correlation analysis has been used in many research fields. Wieleba [10] applied correlation analysis to tribological research, evaluating the coefficient of friction and wear rate of PTFE composite with steel counterface roughness and hardness. Lockwood *et al.* [11] used digital image correlation (DIC) to provide an accurate and fast method of digitally reconstructing fracture surfaces. Ekinci *et al.* [12] investigated the relationship between the motion errors of the axis' carriage and the guideways' geometric errors both mathematically and experimentally. The analysis and experiments just for the bearings location and stiffness, guideway and static equilibrium, do not research the relationship between the geometric error and performance of guideway and flatness of the workpiece, and does not identify the guideway errors.

From the above, the previous work just measured or modeled the single component error of a machinery tool. These methods will introduce some errors. The analytical results are different from the actual values. Some researchers have analyzed and identified the motion errors of machine tools from several measured results, but they cannot identify the dominant error from the flatness of workpieces.

Multi-body theory is a theory developed several decades ago and used for analyzing complex mechanical systems with movement errors [13]. It can be generalized and used to describe complex mechanical systems. Also, both various factors affecting the systems and the mutual coupling relationship can be fully considered. During the early design stages, the kinematic behavior of machine tools can be simulated using multi-body simulation (MBS) tools and a rough estimation [14] can be obtained using rigid bodies. The simulation enables design engineers to make a preliminary and quick prediction of the kinematic behaviors so as to estimate the effects of the variations of the parameters in the model. The rigid coupled multi-body simulation tool can be used to simulate the kinematic behaviors of machine tools while the control loops of the drives are considered [15–17]. Since a machine tool is composed of various parts and it is a multi-body system, multi-body theory can be applied to study its behaviors.

This paper proposes a model for identifying carriage errors of a multi-body system, computes the cross-correlation between the carriage errors and the machining accuracy of a workpiece, as well analyzes and identifies the error sources of the carriage. Straightness and squareness of the carriage are measured and calculated. From the fitting equations, if the errors are given, then a cylindical workpiece is machined and the flatness is measured. Hence, the dominant error sources of the carriage can be deduced by performing the cross-correlation analysis on both the simulation results obtained from the model and the actual data obtained from measuring the flatness of the workpiece. The cross-correlation results show that the impact factor of each error of the carriage on the flatness of the workpiece and the main impact factor of the error from the carriage can be identified.

## 2. Analysis Theory

### 2.1. Multi-Body System and Translated Matrix

**Figure 1.**
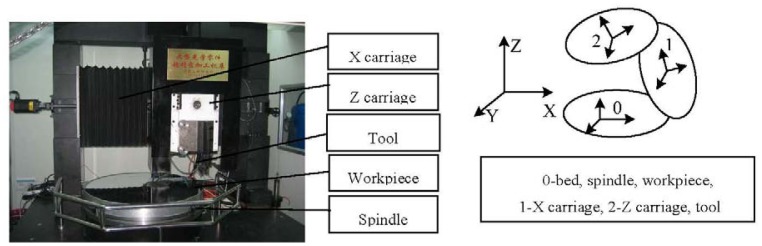
The researched machine tool. (**a**) The structure; (**b**) The topology.

In this session, the multi-body theory is used to model the errors of the carriage. In the multi-body system, topology and low-order array are used for describing the relationship of the physical body. Topology is a major area in mathematics. It is a subject that studies the preservative properties of continuous deformations of the objects, such as the deformations due to stretching without tearing or gluing. This subject has overlapped with geometry and set theory, such as space, dimension and transformation. It is used to describe a family of sets that have certain properties and are used to define a topological space which is a basic object of topology. Figure 1(a) shows the structure of a machinery tool in the laboratory. It includes X and Z axes which are supported by a bridge. The topology of a machinery tool is obtained from its structure. As shown in Figure 1(b), body 0 includes a bed, a spindle and a workpiece. Body 1 is the X carriage. Body 2 includes the Z carriage and a tool. The low-order array is calculated as follows:

(1) $$\{\begin{array}{c} L^{n}(j)=L(L^{n-1}(j))=S\\ L^{0}(j)=j\\ L^{n}(0)=0 \end{array}$$

where *L* is the operator of the lower-order and *S* is *n* lower-order of body *j* when *L*(*j*) = *S*, *S* is the adjacent lower body of the body *j*, and *j* is the adjacent higher-order body of body *S*. In the multi-system, the body which is connected to the ground is fixed. It is called the inertia body. The coordinate of each body is the sub-coordinate in the coordinate system of the inertia body.

The total error motion of the *i^th^* and *j^th^* body is a combination of the rotational and translational errors. For the motion of the body *i*, it rotates anticlockwise by the angle *α_ij_* about the *X* axis first. Then, it rotates anticlockwise by the angle *β_ij_* about the *Y* axis. Finally, it rotates anticlockwise by the angle *γ_ij_* about the *Z* axis. At the same time, it translates *x_ij_* along the *X* axis first. Then, it translates *y_ij_* along the *Y* axis. Finally, it translates *z_ij_* along the *Z* axis. The actual position of the body *j* with respect to the reference frame can be obtained by multiplying the coordinate transformation matrix. The transformation matrix of the body *i* with respect to its adjacent body *j* is given below:

(2) $$T_{ij}=\left[\begin{array}{cccc} 1 & 0 & 0 & 0\\ 0 & \cos\alpha_{ij} & -\sin\alpha_{ij} & 0\\ 0 & \sin\alpha_{ij} & \cos\alpha_{ij} & 0\\ 0 & 0 & 0 & 1 \end{array}\right]\;\left[\begin{array}{cccc} \cos\beta_{ij} & 0 & \sin\beta_{ij} & 0\\ 0 & 1 & 0 & 0\\ -\sin\beta_{ij} & 0 & \cos\beta_{ij} & 0\\ 0 & 0 & 0 & 1 \end{array}\right]\;\left[\begin{array}{cccc} \cos\gamma_{ij} & -\sin\gamma_{ij} & 0 & 0\\ \sin\gamma_{ij} & \cos\gamma_{ij} & 0 & 0\\ 0 & 0 & 1 & 0\\ 0 & 0 & 0 & 1 \end{array}\right]\;\left[\begin{array}{cccc} 1 & 0 & 0 & x_{ij}\\ 0 & 1 & 0 & y_{ij}\\ 0 & 0 & 1 & z_{ij}\\ 0 & 0 & 0 & 1 \end{array}\right]$$

where *α_ij_*, *β_ij_*, *γ_ij_* are the Eulerian angles of axis *X*, *Y* and *Z; x_ij_*, *y_ij_*, *z_ij_* are the linearity displacement in axis *x*, *y* and *z*; subscript *i* and *j* is the body number. The transformation matrix between two random body *i* and *k* is:

(3) $$T_{ik}=\prod_{L^{n}(k)=i}^{n=1}T_{L^{n}(k)L^{n-1}(k)}$$

### 2.2. Components Error of the Machinery Tool

A rigid solid body has six degree of freedom [18], three translational errors and three rotational errors (roll, pitch, and yaw) for determining its location. When the slide moves along any axis, five degrees of freedom are limited in the space. In this paper, the errors of two carriages of the machinery tool shown in Figure 1(a) are modeled based on the homogeneous coordinate transformation. The description of the carriages errors is shown in Figure 1(b). Since the effect of the carriages errors on the machining results has been just considered as well as the spindle and the workpiece are shown together with the bed, they are fixed with the ground. The Z carriage and the tool are shown together. That is, the total error includes the Z carriage error. The tool's coordinate is an ideal condition, but it's error is ignored. Here, the corresponding errors of the X carriage are shown with the subscript 1, and the Z carriage ones are expressed with the subscript 2. The positional errors Δ*y*_1_, Δ*z*_1_, Δ*x*_2_ and Δ*y*_2_ are the translational errors. Δ*α*_1_ and Δ*γ*_2_ are the roll errors of the carriage, Δ*β*_1_ and Δ*β*_2_ are the pitch errors. Δ*γ*_1_ and Δ*α*_2_ are the yaw errors. Assume that the coordinates of the tool at *P* (*p*_x_, *p*_y_, *p*_z_):

(4) $$P_{A}=T_{1}T_{21}P=\left[\begin{array}{cccc} 1 & -\Delta \gamma_{1} & \Delta \beta_{1} & \Delta x_{1}\\ \Delta \gamma_{1} & 1 & -\Delta \alpha_{1} & \Delta y_{1}\\ -\Delta \beta_{1} & \Delta \alpha_{1} & 1 & \Delta z_{1}\\ 0 & 0 & 0 & 1 \end{array}\right]\;\left[\begin{array}{cccc} 1 & -\Delta \gamma_{2} & \Delta \beta_{2} & \Delta x_{2}\\ \Delta \gamma_{2} & 1 & -\Delta \alpha_{2} & \Delta y_{2}\\ -\Delta \beta_{2} & \Delta \alpha_{2} & 1 & \Delta z_{2}\\ 0 & 0 & 0 & 1 \end{array}\right]\;\left[\begin{array}{c} p_{x}\\ p_{y}\\ p_{z}\\ 1 \end{array}\right]$$

The deviation between the actual coordinate *P_A_* and the ideal coordinate *P* is the volumetric error. That is, the machinery error under the effect of the carriages is:

(5) $$E=P_{A}-P=\left[\begin{array}{c} p_{x}-(\Delta \gamma_{1}+\Delta \gamma_{2})p_{y}+(\Delta \beta_{1}+\Delta \beta_{2})p_{z}+\Delta x_{1}+\Delta x_{2}\\ (\Delta \gamma_{1}+\Delta \gamma_{2})p_{x}+p_{y}-(\Delta \alpha_{1}+\Delta \alpha_{2})p_{z}+\Delta y_{1}+\Delta y_{2}\\ -(\Delta \beta_{1}+\Delta \beta_{2})p_{x}+(\Delta \alpha_{1}+\Delta \alpha_{2})p_{y}+p_{z}+\Delta z_{1}+\Delta z_{2}\\ 1 \end{array}\right]-\left[\begin{array}{c} p_{x}\\ p_{y}\\ p_{z}\\ 1 \end{array}\right]=\left[\begin{array}{c} -(\Delta \gamma_{1}+\Delta \gamma_{2})p_{y}+(\Delta \beta_{1}+\Delta \beta_{2})p_{z}+\Delta x_{1}+\Delta x_{2}\\ (\Delta \gamma_{1}+\Delta \gamma_{2})p_{x}-(\Delta \alpha_{1}+\Delta \alpha_{2})p_{z}+\Delta y_{1}+\Delta y_{2}\\ -(\Delta \beta_{1}+\Delta \beta_{2})p_{x}+(\Delta \alpha_{1}+\Delta \alpha_{2})p_{y}+\Delta z_{1}+\Delta z_{2}\\ 1 \end{array}\right]$$

### 2.3. Cross-Correlation Analysis

The cross-correlation techniques have been widely used in engineering and science, particularly in the fields of measurement and communication [19]. The power of the techniques lies in their ability to eliminate the independent noise and the disturbances naturally occurring in the systems. In this paper, a correlation technique has been demonstrated to constitute a powerful tool for identifying the main impact errors of the carriage system on machinery accuracy. In the signal processing community, the cross-correlation refers to a time-lag function used to measure the similarity of two waveforms. To characterize a correlation between two random variables *x* and *y*, where their realizations are denoted as *x* = {*x*_1_,….,*x*_n_} and *y* = {*y*_1_,….,*y*_n_}, respectively, the correlation coefficient is used to measure the similarity between these two random variables [20]:

(6) $$\gamma_{xy}=\frac{\overset{n}{\underset{i=1}{\text{∑}}}(x_{i}-\bar{x})(y_{i}-\bar{y})}{\sqrt{\overset{n}{\underset{i=1}{\text{∑}}}{(x_{i}-\bar{x})}^{2}}\sqrt{{\overset{n}{\underset{i=1}{\text{∑}}}\left(y_{i}-\bar{y}\right)}^{2}}}$$

### 2.4. Daubechies Wavelet

Daubechies wavelets can describe the details of signals because of their compact support and orthogonality properties. Another advantage of using compact support wavelets is they have fewer degree of freedoms than the others. Daubechies wavelets have enormous potential for the analysis of problems with local high gradients. For constructing Daubechies, the properties of Daubechies wavelets are presented below. A more detailed description can be found in [21]. Like other wavelets, both the scaling function *φx*) and the wavelet function *ψ*(*x*) of the Daubechies wavelets satisfy the following two-scaling relation:

(7) $$\phi (x)=\sum_{i=0}^{N-1}p_{i}\phi (2x-i)$$

(8) $$\psi (x)=\sum_{i=2-N}^{1}{\left(-1\right)}^{i}p_{1-i}\phi (2x-i)$$

Define *p_i_*(*i* = 0,1,…,*N*-1) as the so-called filter coefficients, the scaling function *φ_N_* (where *N* is an even integer) has a support in [0, *N*-1], while the corresponding wavelet *ψ_N_* has a support in [1-*N*/2, *N*/2] with *N*/2-1 vanishing wavelet moments [22]. Different choices of *φx*) and *ψ*(*x*) may have different multi-resolution properties. The scaling functions have compact support.

## 3. Impact of the Carriage Errors on the Flatness of the Workpiece

According to Equation (5), the machinery error caused by the *X* carriage in the *Z* direction can be expressed as:

(9) $$Ez=-(\Delta \beta_{1}+\Delta \beta_{2})p_{x}+(\Delta \alpha_{1}+\Delta \alpha_{2})p_{y}+\Delta z_{1}+\Delta z_{2}$$

where Δ*β*_1_ and Δ*β*_2_ are the pitch error and the squareness error of the carriages, Δ*z*_1_ is the straightness of the *X* carriage, Δ*z*_2_ is the positional error of the *Z* carriage and is ignored here, *p_x_* is the displacement of the tool of the machine tool, *p_y_* = 0 for two-axis machine tool.

### 3.1. Straightness of the Carriage

**Figure 2.**
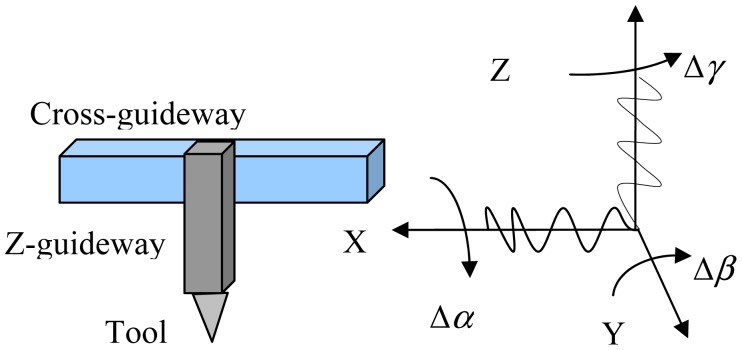
The processing error caused by the straightness error of carriage.

If the *X* carriage has a straightness error Δ*z*_1_(*x*) in the vertical plane, then it will be reflected in the tool path. As shown in Figure 2, the cross carriage is expressed with subscript 1, and Z carriage is expressed with subscript 2, Δ*α*_1_, Δ*γ*_2_ are roll errors of carriage, Δ*β*_1_, Δ*β*_2_ are the pitch errors, Δ*γ*_1_, Δ*α*_2_ is the yaw errors, the guideway is for turning machine tool. In this research, we turn a cylindrical flat. Since the tool fixed on the *z* guideway moving in the *X* direction, the main error due to the pitch error. Δ*β_2_*(*z*) is inducted by the variation of the *x* coordinate in the Z direction, the yaw error Δ*α*_2_(x) is not affect by the flatness of the machinery flat workpiece. The calculated value is ignored. The pitch error Δ*β*_2_(*z*) is obtained by straightness Δ*x*_2_(*z*) of the *Z* carriage. They are expressed by in a polynomial form as follows:

The straightness of the *X* carriage in the vertical plane:

(10) $$\Delta z_{l}(x)=\sum_{k=0}^{n}b_{k}x^{k}$$

The induced yaw error:

(11) $$\Delta \gamma_{1}(x)=\tan^{-1}\frac{dy}{dx}=\tan^{-1}\sum_{k=1}^{n}k\cdot a_{k}\cdot x^{k-1}\approx \sum_{k=1}^{n}k\cdot a_{k}\cdot x^{k-1}$$

The pitch error:

(12) $$\Delta \beta_{1}(x)=\tan^{-1}\frac{dz}{dx}=\tan^{-1}\sum_{k=1}^{n}k\cdot b_{k}\cdot x^{k-1}\approx \sum_{k=1}^{n}k\cdot b_{k}\cdot x^{k-1}$$

Pitch error Δ*β*_2_(*z*) is:

(13) $$\Delta \beta_{2}(z)=\tan^{-1}\frac{dx}{dz}=\tan^{-1}\sum_{k=1}^{n}k\cdot c_{k}\cdot z^{k-1}\approx \sum_{k=1}^{n}k\cdot c_{k}\cdot z^{k-1}$$

The machinery errors caused by the straightness:

(14) $$Ez_{l}=-(\sum_{k=1}^{n}k\cdot b_{k}\cdot x^{k-1}+\sum_{k=1}^{n}k\cdot c_{k}\cdot z^{k-1})p_{x}+\sum_{k=0}^{n}b_{k}x^{k}$$

### 3.2. Squareness of the Carriage

The major causes for squareness errors are as follows:

1. In multi-axis machine tools, the carriages are located on the same structure. There is an angular error between the carriages and the structure of the machine tool. In Figure 2, the *X*-axis and the *Z*-axis are located on the gantry. They will generate squareness errors when they are not perpendicular to each other.
2. When the two axes are not perpendicular due to the upright column tilts forwards or backwards, or due to the right or left when the column base is not horizontal, the two-axis machine of a reference coordinate system will fix the machinery frame to each body in the kinematic chain. Based on the reference Cartesian coordinate systems, the *Y*-reference axes coincide with the actual *Y* machinery axis. Thus, the actual *Y* axis has no angular error or squareness error component. The actual *X* axis has only one angular or squareness error Δ*β_xz_* on the *ZX* plane. The machinery error caused by squareness is:
  (15) $$Ez_{q}=-\Delta \beta_{xz}p_{x}$$

Therefore, the machinery error caused by the straightness and the squareness of the carriage according to Equation (5) is:

(16) $$Ez=-(\sum_{k=1}^{n}k\cdot b_{k}\cdot x^{k-1}+\sum_{k=1}^{n}k\cdot c_{k}\cdot z^{k-1}+\Delta \beta_{xz})p_{x}+\sum_{k=0}^{n}b_{k}x^{k}$$

## 4. Identification for the Carriage Errors

### 4.1. Simulation for the Carriage Errors

**Figure 3.**
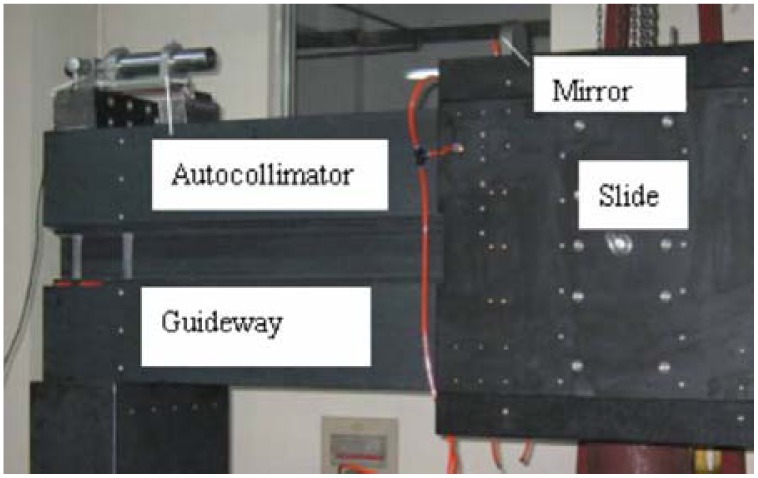
Experimental setup for measurement straightness of cross guideway.

The structure of a machine tool is shown in Figure 1(a). It is a vertical lathe, and it has one cross-slide and one vertical slide. The cross slide belongs to the aerostatic slide type. The tool is set up in the Z (vertical) carriage. The workpiece is supported by the spindle system. Figure 3 shows the experimental setup for the measurement of the straightness of the cross carriage. The photoelectric autocollimator TA80 is mounted on the cross guideway. The measurement range of the device is ±600 arc-s, and the resolution and accuracy are 0.01 arc-s and 0.5 arc-s, respectively. A mirror is mounted on the cross slide. The measurement data is collected with 10 mm intervals. The measured result is 0.3 μm/600 mm. The best polynomial fitting equation of these test data according to Equations (9–16) is:

(17) $$\begin{array}{c} Ez=-2.3\times 10^{-16}x^{5}+3.4\times 10^{-13}x^{4}-1.558\times 10^{-10}x^{3}+2.29\times 10^{-8}x^{2}\\ -5.19\times 10^{-7}x+2.41\times 10^{-4} \end{array}$$

**Figure 4.**
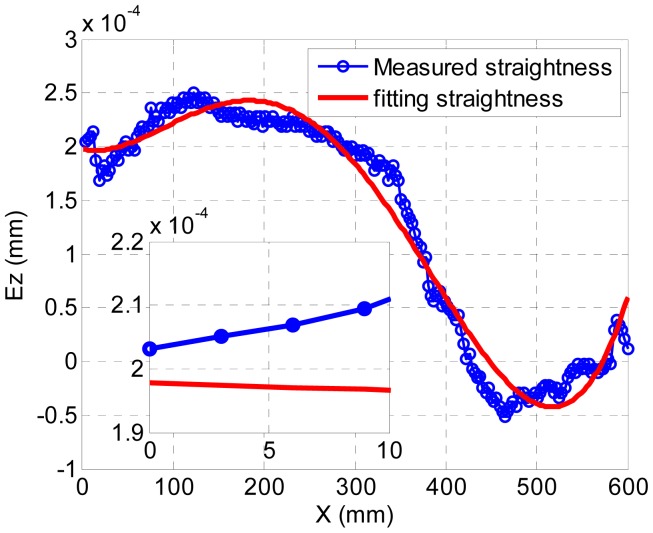
Measurement and fitting curve of straightness.

The results obtained by measuring the data and fitting the curve are shown in Figure 4. The abscissa shows the test range from 0 to 600 mm. The vertical coordinate is the value of the straightness with the unit being mm. It shows that the maximum measured straightness error is 2.5 × 10^−4^ mm, the maximum measured straightness error is 2.48 × 10^−4^ mm and the fitting error is about 0.065 μm. It is approximately at the point of 360 mm test range. Here, the measured straightness is 2 × 10^−5^ mm, and the value of the fitting curve is −4.5 × 10^−5^ mm. The actual tool displacement is from 0 to 10 mm in the X direction as shown in the blue curve of the small figure region in Figure 4.

To verify the effect of the errors of the carriage on the out-of-flatness of the workpiece, an aluminum workpiece with a diameter of 20 mm has been machined by the two-axis lathe. The workpiece is a cylindrical flat, and it is supported by the rotating spindle of the machine tool. The tool is fixed on the Z carriage. The displacement in the horizontal direction is controlled by the cross-slider. The *Z*-directional depth-of-cut of the cutting tool is kept constant, so that a flat surface could be generated. The rotating speed of the spindle is 110 rpm and the cutting depth is 15 μm.

**Figure 5.**
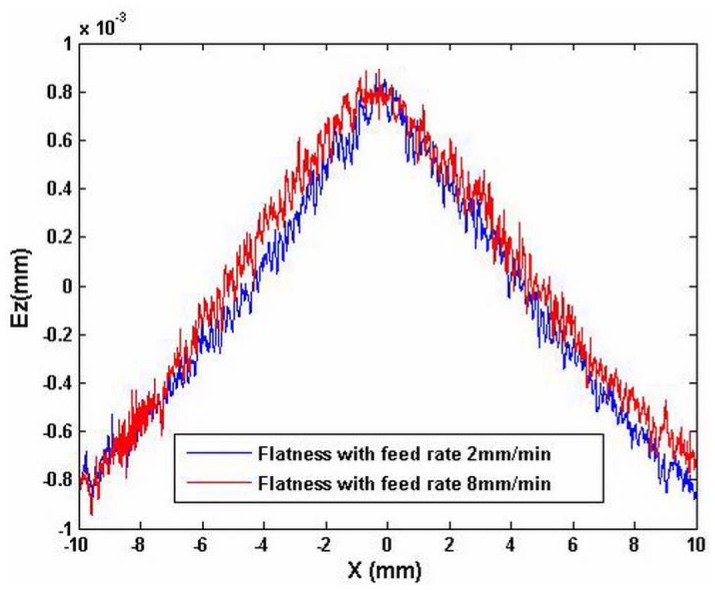
Out-of-flatness of workpiece with different feed rates.

**Figure 6.**
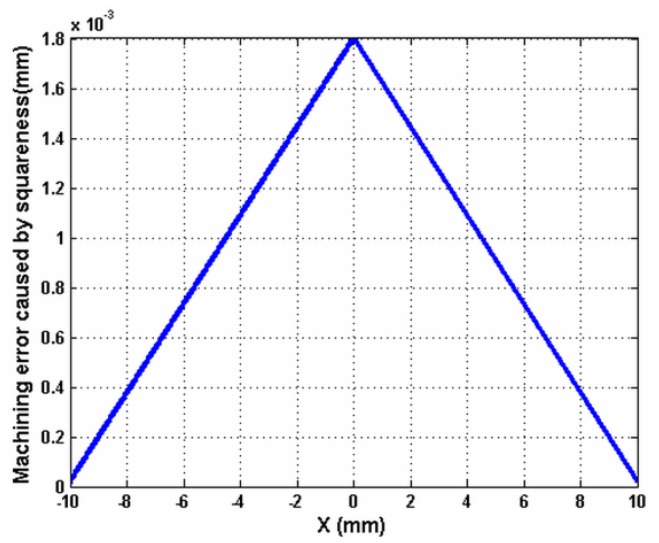
Squareness of guideway.

Figure 5 shows the measured out-of-flatness of the machined surface with a profiler, with different feed rates: 2 mm/min and 8 mm/min. It shows that the out-of-flatness error is approximately 2 μm. It can also be observed that the out-of-flatness profile has a main component. The *p-v* is 2 μm. In addition, there is a lot of waviness. The vibration amplitude with the feed rate 8 mm/min is larger than that with feed rate 2 mm/min. The straightness and the squareness errors of the cross carriage are included in the out-of-flatness profile. There is a relationship among the straightness of the carriage, the feed rate and the spindle rotation speed when both the feed rate and the spindle speed are high. The straightness will be increased. For the squareness error, it is different. It is dependent on the structure of the machine tool. Thus, the errors of the carriage that caused the out-of-flatness have been obtained. The straightness has been tested. The amplitude of the squareness is the same as that of the signal in which the straightness has been removed from the low-frequency test results. The simulation based on Equation (15) is shown in Figure 6. It shows the squareness between the cross carriage and the axis line of the rotation spindle, that is, the normal line of the workpiece supported by the worktable and its value is proportional to the displacement of the tool, it reflects the machining error in machining direction, in ideal situation, it is zero.

### 4.2. Dealing with the Test Result by Daubechies Wavelet

The test result of the machined workpiece includes every error of the machine tool. The errors of the carriage are geometric and the motion errors belong to the low frequency domain. The db1 wavelet is used to decompose the test result first. Then, the test result are expressed as *x*(*t*):

(18) $$x(t)=\sum_{i=1}^{m}x_{i}(t)+\sum_{i=m+1}^{n}x_{i}(t)=s_{1}(t)+s_{2}(t)$$

**Figure 7.**
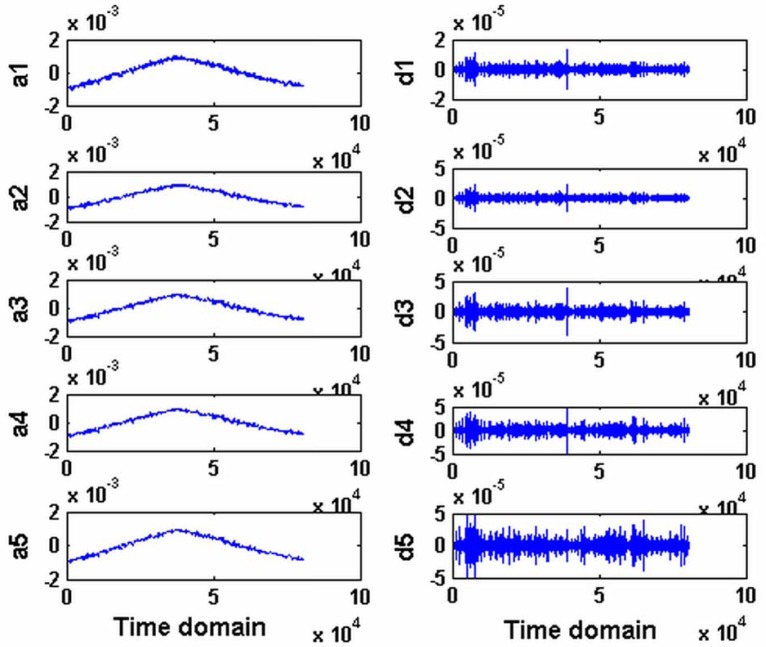
Decomposed result of test by wavelet transformation.

The first part *s*_1_(*t*) constitutes the low-frequency signal and the second part *s*_2_(t) constitutes the high-frequency signal. The signal *x*(*t*) is taken into Equation (18). Then, *s*_1_(*t*) and *s*_2_(*t*) are decomposed from the signal *x*(*t*). In the calculation, the scale is 5 and the test signal is decomposed to five layers. The corresponding wavelet coefficients in different scales are obtained. They represent the error shape in different error bands. The decomposed result is shown in Figure 7. The decomposed low-frequency signal is from *a*1 to *a*5, where *a*1 is the first scale of the test signal and it is very similar to the original low-frequency data. It only includes the low-frequency component. The random error has been removed from it by wavelet methods. It is taken into the correlation analysis with the guideway simulated results discussed in the following. The high-frequency components are from *d*1 to *d*5.

### 4.3. Correlation between Carriage Errors and the Decomposed Signal of the Workpiece Flatness

Here, the carriage errors include the straightness and the squareness. The straightness is determined by measurement. The simulation process of the carriage errors and the decomposed signal of the measured flatness of workpiece are different processes. The shapes of the simulation of the carriage errors and the decomposed low signal (*a*1 in Figure 7) are very similar, in which the error of the carriage is the main error. It cannot be identified just from the test result or only by existing wavelet methods. Here, the correlation analysis is used to identify the errors. The test process is *x*(*t*) = *s*_1_(*t*) + *s*_2_(*t*), where *s*_1_(*t*) is low-frequency component of the test result decomposed by db1 WT, *s*_2_(*t*) is the corresponding high-frequency component. a1 in Figure7 is *s*_1_(*t*). The simulation process of the carriage is *y*(*t*) = *y*_1_(*t*) + *y*_2_(*t*), where *y*_1_(*t*) is the squareness simulation of the carriage, *y*_2_(*t*) is the straightness simulation of the carriage, the correlation between the test result *x*(*t*) decomposed from the low frequency component *x*_1_(*t*) and the squareness *y*_1_(*t*) is calculated, and the straightness *y*_2_(*t*) are analyzed. Therefore, the correlation coefficient *γ_xy_*^m^ and *γ_x_*^1^*_y_m* are obtained by Equation (6) as follows:

(19) $$\gamma_{xy_{m}}=\frac{\overset{n}{\underset{i=1}{\text{∑}}}(x_{i}-\bar{x})(y_{mi}-\bar{y_{m}})}{\sqrt{\overset{n}{\underset{i=1}{\text{∑}}}{(x_{i}-\bar{x})}^{2}}\sqrt{{\overset{n}{\underset{i=1}{\text{∑}}}\left(y_{mi}-\bar{y_{m}}\right)}^{2}}}$$

(20) $$\gamma_{x_{1}y_{m}}=\frac{\overset{n}{\underset{i=1}{\text{∑}}}(x_{1i}-\bar{x})(y_{mi}-\bar{y_{m}})}{\sqrt{\overset{n}{\underset{i=1}{\text{∑}}}{(x_{1i}-\bar{x})}^{2}}\sqrt{{\overset{n}{\underset{i=1}{\text{∑}}}\left(y_{mi}-\bar{y_{m}}\right)}^{2}}}$$

where *m* = 1,2, *γ_xy_m* in Equation (19) is the correlation coefficient between the test process *x*(*t*) and the simulation process *y*(*t*) which includes *y*_1_(*t*) and *y*_2_(*t*). *γ_x_*^1^*_y_m* in Equation (20) is the correlation coefficient between the low-frequency component of the measured result *x*_1_(*t*) and the simulation process *y*(*t*).

**Figure 8.**
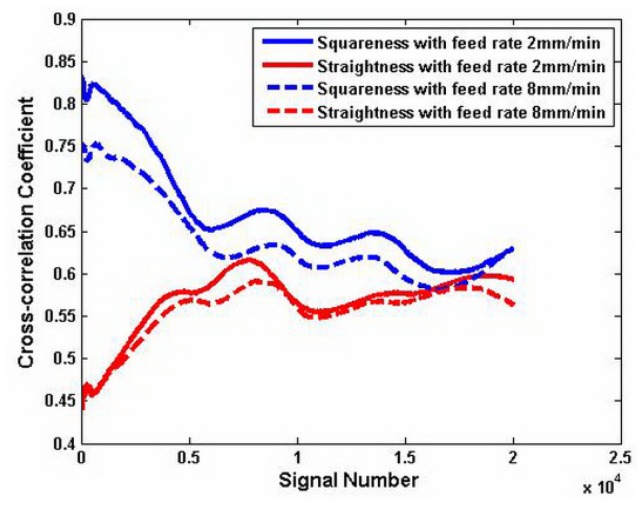
Cross-correlation analysis before using wavelet transformation.

In order to demonstrate the effect of the wavelet transform, the correlation between the carriage errors and test result is shown. The case treated by the wavelet transform and that without treating are compared. Figure 8 shows the results of the correlation of two different test result *x*(*t*) without the wavelet transform. The abscissa presents the signal number. The maximum value is 2 × 10^4^. The longitudinal coordinate is the correlation coefficient and it is in the range of [0–1]. Both the correlation coefficient *γ_xy_*^1^ of the squareness error *y*_1_(*t*) and the test result *x*(*t*) both are in the range of 0.6–0.83. The correlation coefficient with the feed rate 8 mm/min is lower than that with the feed rate 2 mm/min, but it is higher than the correlation coefficient of the straightness with the same feed rate. The correlation coefficient *γ_xy_*^2^ of the straightness error *y*_2_(*t*) and the measured result *x*(*t*) is from 0.45 to 0.6. It is lower than the correlation of the squareness.

**Figure 9.**
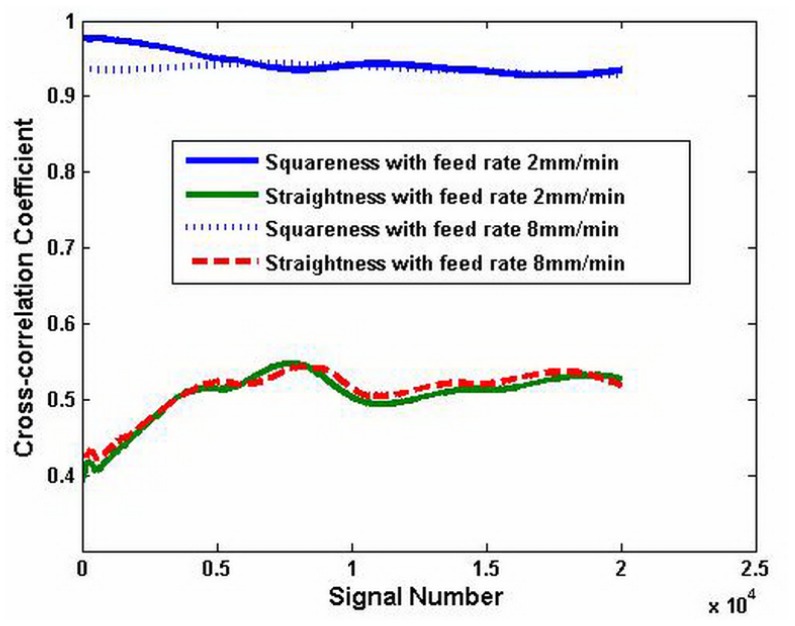
Cross-correlation analysis after using wavelet transformation.

Figure 9 shows the results of the correlation of the low signal test result *x*_1_(*t*) processed by the wavelet transform. The correlation coefficient *γ_x_*^1^*_y_*^1^ of the squareness error and the measured result is close to 1. It means that the squareness error of the carriage is the main error in the test results. The correlation coefficient *γ_x_*^1^*_y_*^2^ of the straightness is similar to that shown in Figure 8. This shows that the measured results need to be processed by the wavelet transform in order to get the ideal signal. It also indicates that the cross-correlation method is valid for identifying the main error of the carriage.

### 4.4. Experimental Verification

**Figure 10.**
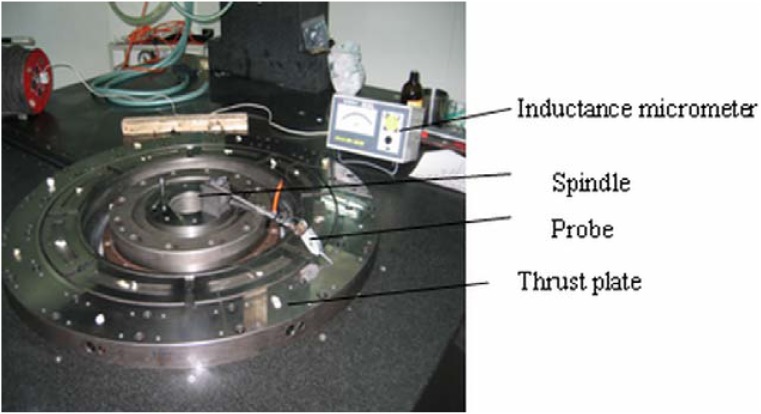
Measurement of squareness between guideway and spindle rotated line.

In the above correlation analysis, it shows that the squareness between the cross carriage and the axis line of the spindle (that is, the normal line of the workpiece shown in Figure 1(a)) is the main error source of the machine tool. Figure 10 shows an indirect measurement of the squareness. It verifies the identification results based on the correlation analysis. An inductance micrometer is placed on the top of the spindle. The probe is in touch with the thrust plate (assume that it is parallel to the cross carriage). It measures the run-out of the spindle. The run-out of the thrust plate at *r* = 0.3 μm is obtained. The largest deflection angle of the spindle can be deduced by *r*. It is assumed that the thrust plate is parallel to the cross carriage in the measurement. In fact, a parallelism error between them is *p* = 2.5 μm. The parallelism error should be considered in the calculation of the deflection angle of the spindle. It is shown in Equation (21). *L* = 1,000 mm is the diameter of the worktable. The amplitude value of the deflection angle is 2.8 × 10^−6^ rad:

(21) $$\theta_{0}\approx \tan(\theta_{0})=\frac{\text{r}+p}{L}$$

Based on Equation (15), the above deflection angle is 2.8 × 10^−6^ rad. The squareness error is Δ*β_xz_* = 2.8 × 10^−6^ rad on the *ZX* plane, *p_x_* is the range of the movement of the slide. It is 600 mm. Thus, the amplitude of the squareness is 1.7 × 10^−3^ mm. It can be verified from Equation (15) that this result is very similar to the simulated value 1.8 × 10^−3^ mm shown in Figure 6.

**Figure 11.**
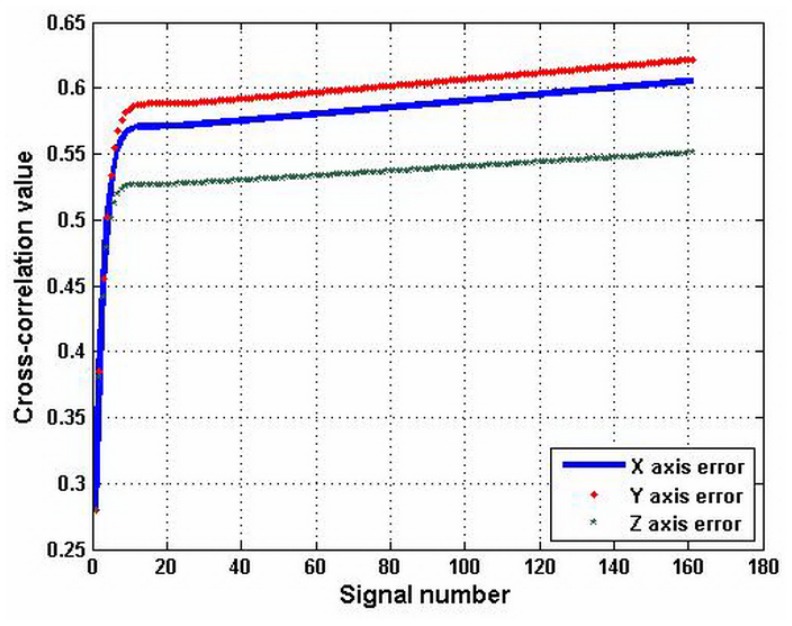
Cross-correlation analysis of the axis errors in [22].

In [22], the simulated errors of the axis *x*, *y*, and z have been obtained. The data in [22] is analyzed by the above correlation method. The result is shown in Figure 11. It shows that the impact factor of the Y error is at the maximum. The impact factor of the Z error is at the minimum. These results are consistent with the simulated results. The method in [22] requires the model and many parameters. It cannot analyze the flatness of workpiece directly. On the other hand, the correlation analysis method is simple and it can analyze the flatness of workpiece directly.

For a large complex structure machine tool, some errors are not easy to test. With correlation analysis, the main impact error can be identified from the flatness of the workpiece of the machine tool. Some of the complicated measurements of the components of the machine tool can be eliminated. This method makes up for the drawbacks of some errors of the machine tools for which we cannot measure the component errors directly.

## 5. Conclusions

This paper applies the cross-correlation method and the wavelet transform to identify the main error of the carriage of a machine tool. The error model which considers the geometry and the motion errors of the carriage system provides an analytical relationship between the flatness error and the carriage system. This method can identify the correlation coefficient between the carriage errors and the flatness of the workpiece. With the wavelet analysis, the identification precision of the test signal is improved. Also, the dominant errors of the carriage and the impact on the flatness of the workpiece are identified. The method can also be applied to the error identification of other machine tool components.
